# Notes and News

**Published:** 1886-11-13

**Authors:** 


### NOTES AND NEWS.

COLLECTED AND COMMUNICATED.

HER Majesty the Queen has always evinced a deep interest in the hospitals of her great empire. Both by purse and personal inspection the Queen has shown her solicitude for the welfare of the sick. Her visit last Friday to the Longmore Hospital for Incurables was thoroughly typical of her general practice. The day, unfortunately, turned out wet, but the loyal spectators loudly cheered the Queen, who was received by Dr. Bell, Dr. Affleck, and other officials of the institution. Her Majesty's visit was not an unexpected one, and many of the patients looked forward to it with lively anticipation. The Queen walked through several of the wards, and took particular notice of some of the patients. To one poor young fellow the Queen addressed some very kind words.

BUT the chief incident in connection with Her Majesty's visit to Longmore Hospital was her notice of the "poetess of the institution," a patient who, despite her incurable malady, rejoices in the name of Madge Fortune. Madge, who seems to be one of nature's poets, could not let so memorable an occasion as a royal visit to Longmore pass without its due celebration in verse, so she wrote a little poem, and as Her Majesty went to her bed, Madge craved and obtained for it her gracious acceptance. Madge Fortune then asked a second favour of her sovereign—would the Queen inscribe her name in her birthday book? The second request was likewise granted, and Madge Fortune is now the happy possessor of the royal autograph. Before leaving, the Queen expressed herself highly satisfied with all she had seen.

A MOTHER'S devotion is a constant quantity in all ranks and classes of society. Lady Dufferin has gone to Lucknow to nurse her son, Lord Clandeboye, who is suffering from an attack of typhoid fever.

A NEW hospital for the town of Hamburg is approaching completion at Eppendorf. Exclusive of the administration block, it consists of eighty distinct pavilions, in extensive grounds. When finished, it is said that this will be the largest and most perfect hospital in Germany.

WE are glad to note the increased interest shown by the employés of the various railway companies in the Hospital Saturday Fund. Up to the end of October, a sum of £257 odd had been received from this source alone.

THE efforts made by the Sunday School Scholars' Fund in aid of the Children's Hospital at Birmingham have resulted in the collection of £340 for that useful charity.

MR. SOAMES, of 34, Dorset Square, N.W., has sent a cheque for thirty guineas to the secretary of the Staffordshire General Infirmary, for the benefit of that institution, enclosed in a letter of grateful thanks for the kindness shown to his little boy, who fell from an express train near Stafford, and was carried to the infirmary for treatment.

THE Sub-Dean has returned the half-guinea prize which was awarded him for his amusing anecdotes, with the request "that the Editor of THE HOSPITAL will dispose of it as he sees fit." The amount has accordingly been placed to the credit of our "Hospital Charity Box Fund." We cordially thank the Sub-Dean for the second set of anecdotes he has sent, and which we shall publish in due course. We shall always be glad to hear from him.

AT the annual general meeting of subscribers to the Wallingford Cottage Hospital, held on the 25th ult., Mr. H. B. Williams-Wynn was unanimously re-elected chairman.

THE funds of the Devon and Exeter Hospital have recently been augmented by a donation of one hundred guineas from an Exeter gentleman, who did not wish his name published. This generous individual has already contributed the same amount to each of six other charitable institutions in Exeter.

SO large a number of patients have of late sought relief at St. Paul's Eye and Ear Hospital, Liverpool, that it has been decided to engage an additional assistant surgeon, and proposals for the increase of the medical staff, and the extension of the buildings, are under consideration.

AN entertainment was recently given at Llandudno, in aid of the local Cottage Hospital, Mr. Dixon, of the Inner Temple, being the chief performer, and grateful thanks are due to him and his assistants for the generous service they have thus rendered to a useful medical charity.

A BAZAAR is to be held at the Royal Concert Hall, Hastings, on the 7th, 8th, and 9th of December, in aid of the rebuilding fund of the East Sussex, Hastings, and St. Leonard's Hospital.

ON the 27th ult. a very successful concert was given in the Bootle Town Hall, in aid of the new Bootle Hospital, under the auspices of a working men's committee, formed of men from the various steam-ship companies at North-end. At a similar entertainment last year the sum of £68 was realised.

NEARLY 2,000 patients have undergone treatment at the West of England Eye Infirmary, Exeter, during the past twelve months, and absolute cures have been effected in 500 of these cases. We are glad to learn that the financial state of the infirmary is entirely satisfactory.

OWING to the munificent gift of the late William H. Vanderbilt, plans have been prepared for a three-story and basement clinical hospital, which is to be erected at Tenth Avenue and Sixtieth Street, New York, by the College of Physicians and Surgeons. The structure will be fireproof, and will cost 130,000 dols. The architecture will be the same as the other buildings of the college group in Fifty-ninth Street.

THE following list of the gross amounts collected since the establishment of the Hospital Sunday movement in Birmingham in 1859 cannot fail to prove of interest.

| | | | | | |
|---|---|---|---|---|---|
| 1859 | General Hospital.. | £5,200 | 1873 | Amalgtd. Charities | £5,370 |
| 1860 | Queen's Hospital.. | 3,433 | 1874 | General Hospital .. | 5,579 |
| 1861 | Amalgtd. Charities | 2,953 | 1875 | Queen's Hospital .. | 5,810 |
| 1862 | General Hospital.. | 3,340 | 1876 | Amalgtd. Charities | 5,283 |
| 1863 | Queen's Hospital.. | 3,293 | 1877 | General Hospital .. | 5,280 |
| 1864 | Amalgtd. Charities | 3,178 | 1878 | Queen's Hospital .. | 6,482 |
| 1865 | General Hospital .. | 4,256 | 1879 | Amalgtd. Charities | 5,159 |
| 1866 | Queen's Hospital .. | 4,133 | 1880 | General Hospital .. | 4,886 |
| 1867 | Amalgtd. Charities | 3,654 | 1881 | Queen's Hospital .. | 4,700 |
| 1868 | General Hospital .. | 4,253 | 1882 | Amalgtd. Charities | 5.195 |
| 1869 | Queen's Hospital .. | 4,469 | 1883 | General Hospital .. | 5,145 |
| 1870 | Amalgtd. Charities | 4,111 | 1884 | Queen's Hospital .. | 4,674 |
| 1871 | General Hospital .. | 4,886 | 1885 | Amalgtd. Charities | 4,773 |
| 1872 | Queen's Hospital .. | 5,192 | 1886 | General Hospital .. | 4,000 |

The amount for 1886 is estimated, as the collections are not yet closed. We have commented in another column upon the falling off in the amounts received of late years from these collections, despite the immense increase in the population.

THE charities of Lancashire seem in luck's way. Miss Alice Lowe, a Blackpool lady, who died recently, has left £4,500 to the Bolton Infirmary, £1,000 to found a similar infirmary for Blackpool, and £500 to the Royal Albert Asylum, Lancaster.

THE Misses Briscoe, of Bohemia House, Hastings two ladies who are well known in local circles for their great munificence, have just sent a cheque for £500 to the building-fund of the new hospital which is now in course of erection at White Rock. The Magdalen Charity has also given £200 to the new hospital at Hastings.

THE Australian papers report the following sad accident in connection with an architectural competition. A new wing, to be known as the Geneviève Ward wing, is to be added to the Maternity Hospital at Melbourne. Architects were invited to send in competitive designs, and a premium of £100 was offered for the one selected. One of these, bearing the motto "Isolation," was selected by the judges. Two days before this decision was arrived at, Mr. Snell, the author of the successful design, committed suicide, driven mad by destitution.

THE 105th anniversary of the foundation of the Nottingham General Hospital was celebrated on Friday last. The Governors and subscribers of the institution met in the grand jury room at the Shire Hall, and walked in procession to St. Mary's Church, the Mayor (Alderman Lambert) and the Sheriff wearing their robes and chains of office. The Rev. Canon Fleming, Chaplain-in-Ordinary to the Queen, was the preacher, and the collection amounted to £233. We are indebted to Miss Rimington for an account of the annual meeting, a notice of which is deferred till next week.

THE maid who burnt a MS. volume of Carlyle's *French Revolution* has at length been paralleled by the achievement of a member of the other sex. The authorities of the County Lunatic Asylum at Upton, near Chester, recently decided to hold a harvest festival in the chapel of their institution. Friends sent welcome presents of flowers, cereals, and vegetables, and the work of decoration went on apace. Late on the Saturday night preceding the Sunday when the harvest festival was to be held, the decorators desisted from their labours and retired to the dormitories of the Upton Asylum, well satisfied with the appearance of the chapel. But, before leaving, they called in a man to remove the litter about the place, and to make all tidy for the Sabbath. Some of the decorators rose early on the Sunday morn, and proceeded to the chapel to take a peep, before service, at their previous night's labour. One may judge the amazement of the officials of the Asylum when nothing but the plain walls of the little chapel met their gaze—the offerings of Flora and Pomona were gone! The man who had been commissioned to sweep up the place was sent for and interrogated. "You told me," he said, "to clear away all the litter, and I did, all of it; I pulled down the litter off the pulpit and places, and put it out in a heap." Then the man conducted the officials of the Upton County Lunatic Asylum to the spot where he had deposited the "litter," and there, sure enough, it lay. "But where is the fruit?" asked one of the party. "Well," he replied, "it seemed a pity to chuck them nice grapes and sich away, so we eat 'em." We are not informed whether the officials of the Upton County Lunatic Asylum have thought it advisable to receive this individual within the walls of their institution.

THE existence of a "Trained Nurses' Club," where those engaged in nursing can meet to read about or talk over their work, will doubtless be welcome intelligence to many of our readers. A few months ago the Matrons' Aid Society, originally founded in 1882 for the use of midwives only, decided to consider trained nurses eligible for election in future, and to add the words, "and Trained Nurses' Club," to its original title. There is an entrance-fee of 2*s.* 6*d.*, and a yearly subscription of 5*s.*, which entitles the subscriber to the full benefits of the club. The club-room is at 15, Buckingham Street, Strand, and is open on Friday evenings from 6 to 10 o'clock. Medical and other papers are provided, and there is a good medical lending library for the use of the members. Full information will be given on application to the Secretary at the above-named address.

HAVING succeeded in obtaining the £3,000 for the building-fund of the proposed new hospital at West Ham, it has been decided to start a sustentation fund, to defray the maintenance expenses. All philanthropists who believe in thrift, and who desire to recognise and encourage the working-classes to help themselves and their families in the hour of sickness, should take an early opportunity of subscribing to the sustentation fund of the West Ham Hospital. Subscriptions may be sent to Mr. F. Mulley, secretary of the West Ham Dispensary.

Bournemouth is determined to turn the forthcoming great jubilee celebration to practical account. The Commissioners have just appointed a committee to consider a proposal which has been made to commemorate the year of jubilee by the erection of a hospital.

---

The late Mr. Thomas Phillips Danson, of Bootle, near Liverpool, has left £1,000 to the Endowment Fund of the Bootle Borough Hospital, and a further sum of £500 to the Liverpool Dispensaries.

---

In reference to the discussion on the registration-fee payment by patients at general hospitals, the following notes may be of interest:—John Howard, in his work on Lazarettos, records that he saw at Guy's Hospital "a woman bring her child, and with tears leave the fee of 2*s.* 9*d.* for the nurse, and 6*d.* for the steward. The foul patients pay 7*s.*—September 17th, 1788." At St. Bartholomew's, he says: "Fees are taken for the admission of patients. For clean patients, 2*s.*; viz., 1*s.* for the sister, 6*d.* to the nurse, 6*d.* to the beadle; for foul patients, £1 5*s.* 8*d.*, viz., 5*s.* for flannels, 18*s.* 8*d.* for two months' subsistence, at 4*d.* per day, and 2*s.* ward dues."

---

It appears from an article in the *Hastings Chronicle* that the inhabitants of Hastings and St. Leonards are anxious that the world should know through the London papers that Hastings is keeping pace with the times, by the erection of a hospital with circular wards for the accommodation of its patients. We understand that the whole of the money for the new buildings has not yet been obtained, and that those who take an interest in hospitals, and desire the newest form of hospital construction to be extended, will have an opportunity of contributing something of their abundance to a useful and much needed institution.

---

It is often said that the working-class do not contribute adequately to the support of the hospitals. Whether this statement is well founded or not, it is certain that the contributions of the working-classes to hospitals in all parts of the country to-day, as compared with twenty years or even ten years ago, have increased by leaps and bounds. Thus the Burton Infirmary received last year £813 from the Infirmary Saturday collections, and an even larger sum the year before. At Leicester, the treasurer of the Leicester Infirmary gratefully acknowledges the receipt of £1,478, as the result of the Hospital Saturday collections just completed. Burton has a population of 39,288, whilst that of Leicester amounts to 122,376. Are the men of Burton more generously disposed than their *confrères* at Leicester, or how is the difference to be accounted for?

---

Complaints are not infrequently heard of the ingratitude shewn by the poor to those who endeavour to lessen the troubles and alleviate the sufferings of their lives. Few private benefactors, and perhaps still fewer public institutions, have been without experience of what may almost be considered as an absence of sense, rather than wilful ingratitude, in the labouring classes. Not long ago, the wife of the Secretary of one of the best-known Lying-In Hospitals of the metropolis received and opened in mistake, (a mistake caused by similarity of name), the letter we give below. This remarkable epistle was written by the husband of a woman who had been delivered in the hospital of a still-born child, for the burial of which she had been informed that, in accordance with the rules of the institution, she must pay the sum of 5s. 6d. She communicated with her husband on this point, and hence the letter we now quote in full, only suppressing names:

Saturday, Oct. —

"My dear Nell.—I hope your coming out this morning But dont you pay the 5/6 that They wont, They cant keep you there. I was talking to Old—— last night, and He told me that They cant make us pay so dont you pay. dont you get fringhgnd of them. Tell 'em you cant pay it They wont keep you there. Now mind what I tell you Dont pay it. Because that will get your Dress out, keep your few shillings till you comes Dont Be a d— fool and pay it. Because they wont keep you there, So good morning

My Dear Duck

Mind I whouldent take the trouble to write if I thought you whould pay so mind."

---

The woman, either persuaded by her husband's arguments, or frightened by his implied threats, did not pay, and was discharged on her recovery in due course. What is to be said or thought of the individual who could write such a letter as this? There is no expression of gratitude, nor word of thankfulness for his wife's restoration to health. His one idea is to avoid paying for the decent burial of his own child, and to put to every possible expense the authorities, by whose skill and kindness his wife has been cured. Does it not seem as if there was some physical defect, something wanting as it were, in the constitution of such a person?

---

We have just received a copy of the second edition of the *History of Queen Charlotte's Lying-in Hospital*, written by Mr. Thomas Ryan, the able Secretary of that institution. In a short supplementary chapter which Mr. Ryan has added to this issue of his most interesting work, some light is thrown upon the hitherto doubtful situation of the hospital about eighty years ago, and particulars of the recent enlargement and improvements are given. Their Royal Highnesses the Princess of Wales and Princess Christian have been graciously pleased to accept copies of Mr. Ryan's book, which is admirably got up, and well supplied with plans and views of the hospital at different periods of its existence.

---

In his introductory lecture of the present session at the Richmond Hospital, Dublin, Dr. Nugent said, "Fever is *par excellence* the disease of Ireland. The record of the history of this country can be almost as continuously followed through the details of the successive fever epidemics which have decimated its population, as it can through those of its perpetual political and social disturbances." Referring to the Hardwicke Hospital, the lecturer said that the facilities for studying fever there afforded to hospital students were unparalleled by any other clinical hospital in Ireland.

